# Supplementary material for: Graph diffusion distance: Properties and efficient computation
Source: PLoS One. 2021 Apr 27;16(4):e0249624. doi: 10.1371/journal.pone.0249624 (PMC8078789; doi:10.1371/journal.pone.0249624)
Supplement: Attachment — Submitted filename: 2020_PONE_D_20_13130.pdf [file pone.0249624.s002.pdf]

Graph diffusion distance: Properties and efficient computation
PLOS ONE Reviewer Report
October 26, 2020

Article summary

This article presents an in depth look at the Graph Diffusion Distance (originally introduced by Hammond et al. in 2013), insights into its computation, and proofs about its optimization. This is a distance based on the differences between the Laplacian exponential matrices of pairs of graphs, under certain constraints. The authors really take a thorough approach to characterizing this distance measures, and as such, the article reads like quite a comprehensive piece.

Review summary

This article is great. I have been working in the graph distance world for several years now, and I found this contribution to be quite thorough. Whenever I had a "but what about..." question, the authors seemed to answer it in the text right away. As with all graph distance papers, however, there are always more questions to be asked, and I do have a few that I would like the authors to address. Apart from these minor notes, I would like to see this work published in *PLOS ONE*.

I. General comments and requests

1. Graph lineage vs graph ensemble

1. I think the notion of graph lineage is interesting, though I must admit it is the first time I've been exposed to it. For a network science oriented reader, I think there should be slightly more details about what a graph lineage is.

2. At the moment, there is the brief description on page 6, page 28, as well as Figure 2, but I would prefer not to have to refer to reference #2 to better grasp the concept. One idea is to include more references to specific graph lineages throughout the literature, (for example, are Cayley Trees or other generative models of growing networks good examples of lineages?). Similarly, use the the four examples from Section 7.1 as explanatory tools in the description on page 6, as they can offer more intuitions to the reader earlier on.

3. Another idea: From the perspective of a network scientist, I find the idea of a graph ensemble to be much more familiar, so situating the definition of graph lineage might make more sense if done through that lens.

2. This work is quite strong, and it really makes me want to apply these measures. When (or before) this is published, please include a link to publicly available code.

1. Section 6.3 describes the implementation, but as this work describes an exciting family distance measures, readers would greatly appreciate the chance to try it out for themselves.

2. One specific example of this is in Section 7.3.2, where intra- and inter-lineage distances are discussed. This reminds me of recent work discussing a number of *within-ensemble graph distances*[1] (arXiv: `https://arxiv.org/abs/2008.02415`), where the authors measure the distance between pairs of graphs sampled from a given ensemble. I was hoping

to test the distance measures here in a similar way to get a sense of whether they capture certain structural properties of a given ensemble (e.g. critical transition at $\langle k \rangle = 1$ in $G_{(n,p)}$).

II. Specific comments and requests

1. Some entries for Classification in Table 1 are empty. Please make more clear why this is intentional in the caption.

2. In Section 1.1.1, I believe this is the first use of n_1 and n_2—please just add a note after "adjacency matrices $A^{(1)}$ and $A^{(2)}$" something along the lines of "with n_1 and n_2 number of vertices, respectively".

3. Section 2.1, page 7: "very late times the distribution of vertex-visits converges to the uniform distribution for each connected component of the graph" – is this still true for weighted graphs? Or is perhaps "stationary state" better than uniform here?

4. Throughout: If possible, I would prefer a different notation than D^2 to refer to the new distance measure being introduced. I kept second-guessing if I missed some notation explanation. I understand this might be a large change, so if the authors would prefer to keep this, please emphasize the rationale for this notation earlier on.

III. Minor comments and non-urgent requests

1. Figure 1: The tick labels on the horizontal and vertical axes are tiny—if possible, please increase the size.

2. Page 32: "to justify defining graph limits in this way" I think here and in nearby next it might be best to continue to emphasize that this definition is a *variant* of the graph limit definition.

3. If interested: I noticed that an earlier version of this work included slightly different figures (e.g. Figure 6 in `https://arxiv.org/abs/1909.04203`): some of these are quite nice, and I would not object to seeing them in this manuscript. It is already quite a long piece, and periodically adding helpful, if possibly redundant, figures could really broaden the reach.

References

1. Hartle, H. *et al.* Network comparison and the within-ensemble graph distance. *Proc. Royal Soc. A* DOI: 10.1098/rspa.2019.0744 (2020).
