# Supplementary material for: Graph diffusion distance: Properties and efficient computation
Source: PLoS One. 2021 Apr 27;16(4):e0249624. doi: 10.1371/journal.pone.0249624 (PMC8078789; doi:10.1371/journal.pone.0249624)
Supplement: Attachment — Submitted filename: PLOS ONE REVIEW RESPONSE.pdf [file pone.0249624.s003.pdf]

Graph diffusion distance: Properties and efficient computation
***PLOS ONE* Review Response**
November 29, 2020

We thank both reviewers for their thorough and insightful comments. A theme of both reviews was the need for more detailed applications of our work; we hope the numerical experiments and additional examples in the revised version will satisfy these requests. A point-by-point response to each review follows. A note to reviewers and the editor: as a result of these revisions some section and figure numbers have changed. In this document we will make clear whether each number is the new or old label.

Review 1

Article summary
This article presents an in depth look at the Graph Diffusion Distance (originally introduced by Hammond et al. in 2013), insights into its computation, and proofs about its optimization. This is a distance based on the differences between the Laplacian exponential matrices of pairs of graphs, under certain constraints. The authors really take a thorough approach to characterizing this distance measures, and as such, the article reads like quite a comprehensive piece.

Review summary
This article is great. I have been working in the graph distance world for several years now, and I found this contribution to be quite thorough. Whenever I had a “but what about...” question, the authors seemed to answer it in the text right away. As with all graph distance papers, however, there are always more questions to be asked, and I do have a few that I would like the authors to address. Apart from these minor notes, I would like to see this work published in *PLOS ONE*.

I. General comments and requests
1. Graph lineage vs graph ensemble

1. I think the notion of graph lineage is interesting, though I must admit it is the first time I’ve been exposed to it. For a network science oriented reader, I think there should be slightly more details about what a graph lineage is.

   We have amended the definition of graph lineage to include several examples as well as link forward to the later discussion of these examples in the context of graph diffusion distance. We have also included a more detailed definition, removing the reference [2] as it is no longer relevant.

2. At the moment, there is the brief description on page 6, page 28, as well as Figure

2, but I would prefer not to have to refer to reference #2 to better grasp the concept. One idea is to include more references to specific graph lineages throughout the literature, (for example, are Cayley Trees or other generative models of growing networks good examples of lineages?). Similarly, use the the four examples from Section 7.1 as explanatory tools in the description on page 6, as they can offer more intuitions to the reader earlier on.

See previous note. We have added text to the definition of graph lineage to illustrate some examples, as well as a forward link to the old Section 7 (now Section 5) in the definition on page 7.

3. Another idea: From the perspective of a network scientist, I find the idea of a graph ensemble to be much more familiar, so situating the definition of graph lineage might make more sense if done through that lens.

In our response to Reviewer 2's main concern, we have included a new experiment that uses GDD to compare graph ensembles (see also our response to Reviewer 1's point 2.2 below).

2. This work is quite strong, and it really makes me want to apply these measures. When (or before) this is published, please include a link to publicly available code.

1. Section 6.3 describes the implementation, but as this work describes an exciting family distance measures, readers would greatly appreciate the chance to try it out for themselves.

We have provided code (as well as a very basic README) as supplementary material in this revision. A python package for diffusion distance calculations is forthcoming. The code is also available now at https://github.com/scottcb/DiffusionDistance, which may be updated to be more user-friendly than the attached version.

2. One specific example of this is in Section 7.3.2, where intra- and inter-lineage distances are discussed. This reminds me of recent work discussing a number of *within-ensemble graph distances*[1](arXiv: https://arxiv.org/abs/2008.02415), where the authors measure the distance between pairs of graphs sampled from a given ensemble. I was hoping to test the distance measures here in a similar way to get a sense of whether they capture certain structural properties of a given ensemble (e.g. critical transition at $hki = 1$ in $G_{(n,p)}$).

We thank the reviewer for providing this reference. Motivated by the test you mention, we ran the following quick experiment: By computing the within-ensemble distance for graphs drawn from $G_{(512,p)}$ for $p \in [10^{-6}, 1]$, we show that our implementation of GDD does, as expected, have a local maximum at $\langle k \rangle = 1$, as suggested in the cited work:

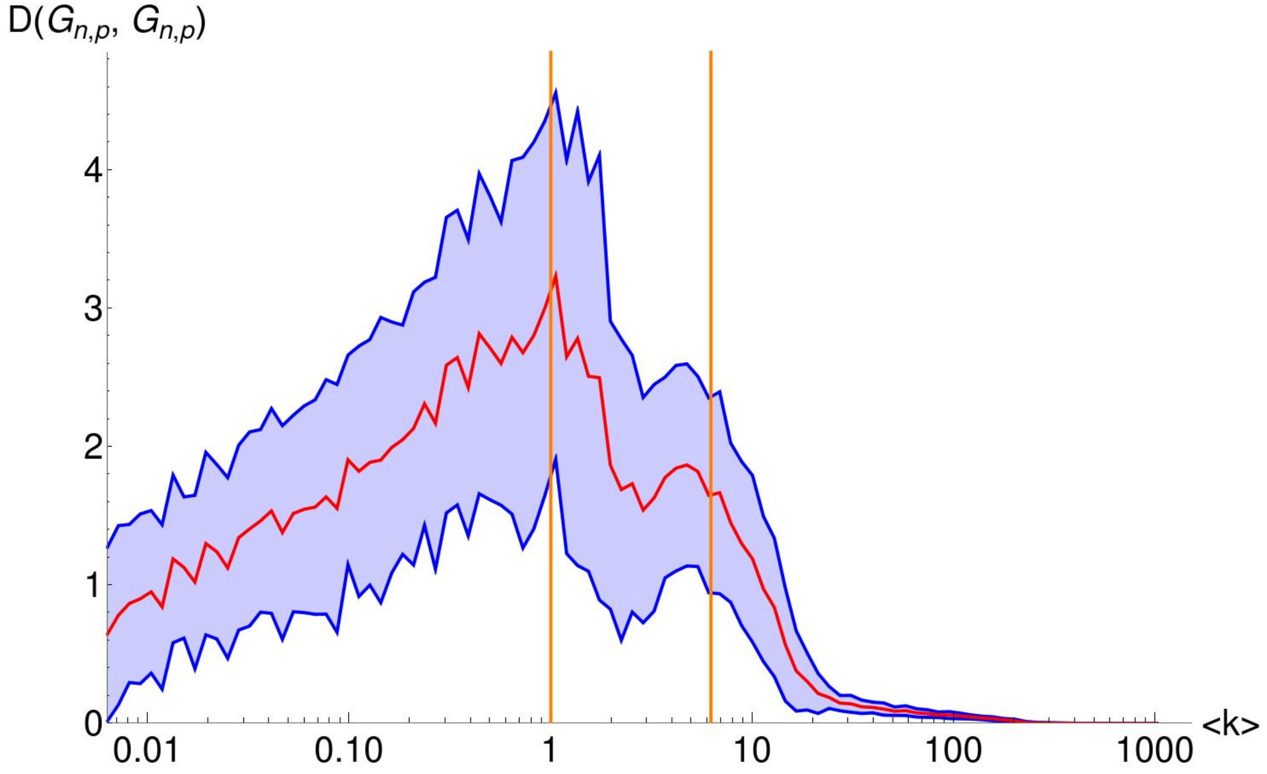

Figure 1: Within-ensemble diffusion distances for graphs drawn from the Erdos-Renyi ensemble on 512 vertices, for various edge occurrence probabilities p. As expected and described in Hartle *et al.*, a critical transition (local maximum) occurs at $p = (1/n)$ (vertical orange line). The second vertical orange line is placed at ln(512), which is the value of <k> where with high likelihood all graphs are a single connected component. Note its proximity to the other local maximum.

We could include this figure in the manuscript, if the reviewer thinks it would substantially improve the paper. For now, we have added a reference to the reviewer's linked paper, and a statement (in Section 5.3.2) to the effect of "graph diffusion distance has been previously shown to capture key structural information about graphs; for example, GDD is known to be sensitive to certain critical transitions in ensembles of random graphs. This is also true for our time-dilated version of GDD."

The new Figure 13 included in the new Section 5.3.5 ("shape analysis") also addresses the question of distances between graphs drawn from graph ensembles - the ensembles in that case being randomly constructed discretizations of 3D mesh objects.

II. Specific comments and requests

1. Some entries for Classification in Table 1 are empty. Please make more clear why this is intentional in the caption.

We have added a short note to the caption of Table 1 to make clear that entries of

the table are blank because their classification is unclear.

2. In Section 1.1.1, I believe this is the first use of n_1 and n_2—please just add a note after "adjacency matrices $A^{(1)}$ and $A^{(2)}$" something along the lines of "with n_1 and n_2 number of vertices, respectively".

We have altered this sentence as suggested.

3. Section 2.1, page 7: "very late times the distribution of vertex-visits converges to the uniform distribution for each connected component of the graph" – is this still true for weighted graphs? Or is perhaps "stationary state" better than uniform here?

"Stationary state" is more correct; we have changed "uniform distribution" to "stationary state".

4. Throughout: If possible, I would prefer a different notation than D^2 to refer to the new distance measure being introduced. I kept second-guessing if I missed some notation explanation. I understand this might be a large change, so if the authors would prefer to keep this, please emphasize the rationale for this notation earlier on.

Many of the proofs (and many of the figures) use properties of the squared distance D^2 rather than D, so we have elected not to make this widespread change. We have added a note at the first distance definition (just below equation 8) to make clear what is meant by D^2.

III. Minor comments and non-urgent requests

1. Figure 1: The tick labels on the horizontal and vertical axes are tiny—if possible, please increase the size.

We have made the axis labels, ticks, and tick labels larger in all subfigures of old Figure 1, and have additionally split this figure so that it is now Figures 3 (old Figure 1a) and 4 (Figures old 1b and 1c). This has made the text proportionally larger, as well as showing the unimodality of the distance function closer in the text to where it is discussed.

2. Page 32: "to justify defining graph limits in this way" I think here and in nearby next it might be best to continue to emphasize that this definition is a *variant* of the graph limit definition.

We have modified the indicated sentence to read "to justify this variant definition of a 'graph limit' ". This is part of broader revisions suggested by Reviewer 2, see below.

3. If interested: I noticed that an earlier version of this work included slightly different figures (e.g. Figure 6 in https://arxiv.org/abs/1909.04203): some of these are quite nice, and I would not object to seeing them in this manuscript. It is already quite a

long piece, and periodically adding helpful, if possibly redundant, figures could really broaden the reach.

We have added several new figures to break up the text, as per this suggestion. These include: a new figure (Figures 1,2, and 5) illustrating several examples of graph lineages, a new figure showing the results of comparing 3D point cloud graphs with GDD (Figure 13), and the specific figure requested by the reviewer (from a prior version of the manuscript; Figure 6 in the revised version).

References

1. Hartle, H. *et al.* Network comparison and the within-ensemble graph distance. *Proc. Royal Soc. A* DOI: 10.1098/rspa.2019.0744 (2020).

Reviewer #2:

I recommend a major revision to address certain concerns about the significance of the results. In particular, it seems that most of the results study how to compute upper bounds on the proposed dissimilarity measures, but I would like to see more explicitly worked out applications of the measures, so that the reader can see why they are useful.

1.) The authors may wish to cite some relevant works on the use of optimal transport to define distances between graphs. See, e.g., GOT: An Optimal Transport Framework for Graph Comparison, by Maretic et al., NeurIPS 2019.

We have added the suggested reference as one of the included distance measures in our taxonomy of other distance metrics in Section 1.1.3., including it in our discussion of Earth Mover Distance (EMD).

2.) The paper mentions a few possible applications. I think that it is important that at least one of these applications be fleshed out (e.g., with experiments) so that we can see the practical utility of the new distance measure. For instance, the application to graph convolutional networks would be good to expand with some experiments.

Since submitting this manuscript, we have submitted and published two works (references [2] and [45]) which use graph diffusion distance and the P matrices calculated in the manner this manuscript describes. One work uses prolongation matrices to accelerate training of flat neural networks, while the other uses the P matrices as pooling operators in a graph convolution framework. We have amended the “Applications” section to mention these works explicitly, with included references.

3.) Regarding the graph limit application, it seems to me that one of the useful aspects of cut distance in defining graph limits as Lovasz and coauthors did is that the limit objects in the cut distance have a nice characterization: they're precisely graphons (i.e., symmetric, Lebesgue measurable functions from the unit square to the unit interval), which have a nice geometric characterization. Furthermore, there is the connection between cut distance and the convergence of subgraph frequencies. Do any such properties hold for the distance proposed in this paper? It seems insufficient that the authors just point out that one can define graph limits as limits of Cauchy sequences of graphs, because that can be done for absolutely any metric between graphs.

They do give some intuition regarding possible advantages of their distance over the cut distance, but I'm not sure that they really follow through with this and prove theorems that make this concrete. For instance, can they formalize their intuition to exhibit certain pairs of growing graph sequences that converge to the same limit in cut distance but diverge with respect to their distance?

This is the case for all sparse graphs (all sparse graph sequences, by which we mean those with fewer than $\Omega(n^2)$ edges as in reference [42], converge to the zero graphon). We have added a note to this effect, and a brief reference to the Benjamini-Schramm framework as well as another work on limits of sparse graph sequences.

Along the same lines, they should compare with the convergence results in the Benjamini-Schramm framework and other notions of convergence for sparse graph sequences. It might be that their distance metrizes the topology corresponding to one of these other convergence notions.

It is the opinion of the authors that the geometric characterization and subgraph frequency suggestions given by the reviewer constitute material for an entirely new research project and resulting paper; while we agree that the proposed approach has merit, we affirm that our paper stands on its own without this possible additional breakthrough which would be an achievement beyond the scope of our work. Our paper's focus is on the properties of Graph Diffusion Distance, as well as a novel numerical algorithm which computes the GDD efficiently. We demonstrate empirically that GDD is useful in shape analysis as well as other machine learning settings. It is the opinion of the authors that these results should not be delayed in favor of further research into the differences between GDD and cut-distance, which may not produce a result of the form requested.

However, in the interest of addressing the points made by Reviewer 2, we have made several changes to Sections 5.3.3 ("Graph Limits"). We have added text explaining why the definition of Lovasz does not extend to sparse graphs, and a citation to the original paper of Benjamini and Schramm introducing sparse graph limits. By doing so, we have made it more clear that the work, as it currently stands, does not thoroughly develop the idea of geometric GDD limit objects, and therefore is not directly comparable

to Lovasz and Benjamini/Schramm. It is true as the reviewer says that a metric space is sufficient to define Cauchy convergence, and one can complete a metric space to include the limit objects. So, are the limit objects under our particular definition of distance useful in some way? We do not have a geometric characterization like graphons for cut distance, and we see no immediate prospect for getting one, but we do provide empirical evidence of the potential utility in Section 5.3.3, Figure 11, and the new Figures 12 and 13.

Figures 12 and 13 also address Reviewer 2's request to flesh out some of the applications of GDD, by demonstrating that GDD may be used to calculate distances between 3D point clouds derived by randomly sampling meshes. These distances naturally reproduce from which of the original meshes a given graph was sampled.

4.) On page 16, they mention Theorem 7 in the past tense, but Theorem 7 appears much later in the paper.

We have fixed the reference on Page 16, and other parts of the same subsection, to refer to Theorem 7 in the future tense.

Comments from the Editor:

1. Please ensure that your manuscript meets PLOS ONE's style requirements, including those for file naming. The PLOS ONE style templates can be found at

https://journals.plos.org/plosone/s/file?id=wjVg/PLOSOne_formatting_sample_main_body.pdf and

https://journals.plos.org/plosone/s/file?id=ba62/PLOSOne_formatting_sample_title_authors_affiliations.pdf

This manuscript was prepared using the official PLOS style files for LaTeX. We have renamed all figure files as requested.

2. Please ensure that you refer to Figure 5 in your text as, if accepted, production will need this reference to link the reader to the figure.

The old Figure 5 (now Figure 11) is now referred to in the text at the beginning of the relevant proof (at the top of page 37, beginning of Section 5.3.4).
